# A ‘Mystery Client’ Evaluation of Adolescent Sexual and Reproductive Health services in Health Facilities from Two Regions in Tanzania

**DOI:** 10.1371/journal.pone.0120822

**Published:** 2015-03-24

**Authors:** Zaina Mchome, Esther Richards, Soori Nnko, John Dusabe, Elizabeth Mapella, Angela Obasi

**Affiliations:** 1 National Institute for Medical Research, Mwanza, United Republic of Tanzania; 2 Liverpool School of Tropical Medicine, Liverpool, United Kingdom; 3 Ministry of Health and Social Welfare, Dar Es Salaam, United Republic of Tanzania; University of Washington, UNITED STATES

## Abstract

Unwelcoming behaviours and judgemental attitudes have long been recognised as a barrier to young people’s access to reproductive health services. Over the last decade youth friendly reproductive health services have been promoted and implemented world-wide. However, long term evidence of the impact of these programmes is lacking. We report the results of a large mystery client evaluation of adolescent sexual and reproductive health services in Tanzania, a country that has had a long established youth friendly policy. Forty-eight visits made to thirty-three health facilities were conducted by twelve young people (six in each region) trained to perform three different scripted scenarios (i.e., condom request, information on sexually transmitted infections and family planning). The study revealed barriers in relation to poor signage and reception for services. In addition health workers demonstrated paternalistic attitudes as well as lack of knowledge about adolescent sexual and reproductive health services. In some cases, health workers discouraged young people from using services such as condoms and family planning methods. Lack of confidentiality and privacy were also noted to be common challenges for the young people involved. Intervention strategies that focus on changing health workers’ mind-set in relation to adolescent sexual and reproductive health are crucial for ensuring quality provision of sexual and reproductive health services to young people. The study identified the importance of reception or signs at the health units, as this can facilitate young people’s efforts in seeking sexual and reproductive health services. Likewise, improvement of health workers knowledge of existing policy and practice on sexual and reproductive health services and youth friendly services is much needed.

## Introduction

The reproductive health of African adolescents and their need for youth friendly reproductive health services (YFRHS) has been the focus of significant policy and intervention activity since the turn of the last century [1–4]. Barriers to service uptake have been well described; the characteristics of youth friendly service provision defined and the principle of access to youth friendly reproductive health services (YFRHS) enshrined within the Millennium Development goals [3, 5]. Yet in the decade since the WHO launched its call for youth friendly services, strong evidence of translation of national and international YFRHS policies into widespread practice is lacking [6]. A large-scale cluster randomised study in Tanzania provided an opportunity to evaluate the current state of YFRHS provision in a country where reproductive health policies for young people have been developed and endorsed by government.

The use of ‘mystery client’, ‘simulated patient’, or ‘mystery shopper’ evaluation methods in assessing quality of service provision in developed countries has proven to be reliable, valid, feasible and acceptable [7–9]. The method involves a trained observer posing as a patient who records their experiences of provider encounters. This has been found to be an appropriate method in capturing ‘real life’ accounts of provider performance and environments in which sexual and reproductive health (SRH) services are provided, including in low-middle income settings [10]. Since the providers do not know they are being observed, in most cases the information gathered through mystery client encounters reflects providers’ normal performance [7].

## Background and Study Setting

The Government of the United Republic of Tanzania was one of the first African countries to respond to the WHO call for action for improved reproductive health service provision for young people, setting standards for youth friendly reproductive health services over a decade ago [11, 12]. As part of the Mema kwa Vijana phase 2 (MkV2) programme, YFRHS training was rolled out in all government health facilities in 4 districts (Sengerema, Geita, Kwimba and Missungwi) in Mwanza Region, Northern Tanzania between 2004 and 2007. An evaluation at that time found the YFRHS training to be effective and practicable [13, 14].

The present study forms part of a baseline situation analysis for the IntHEC project, a cluster randomised evaluation of an intervention which aims to address the cultures within health service, school and community settings. The IntHEC project is being implemented in 36 communities in two districts in Mwanza (Sengerema and Magu) and Iringa (Makete and Mafundi) Regions respectively, and has been described elsewhere (Obasi et al,. *forthcoming*). We present the results of a large mystery client study that evaluated the youth friendliness of reproductive health services in a stratified sample of 33 health units from 24 of the 36 trial communities.

## Methods

Three scripted scenarios addressing a condom request, STI symptoms and family planning query were developed with attendant checklists using previous mystery client evaluation protocols and national guidelines [13] (see Table 1). Six male and female young adults per region were selected on the basis of a participatory assessment of presentation and memory skills from a pool of 36 literate, young looking 18–19 year old volunteers from non-trial communities and trained for 5 days in the implementation of the scenarios. At the end of the training three males and three females were selected to proceed with the study in each region. Each community comprised a ward (approximately 6–8 villages) which is a well-defined administrative unit. These were stratified into rural, urban and special risk groups, where the latter included mining, cotton ginnery, forest or fishing communities. One ward from each strata was randomly selected from each trial group (intervention and comparison) per district giving 12 communities per region with the intention to include both health centres (larger and more central) and dispensaries (smaller and more widespread) in the evaluation. A total of 8 health centres and 25 dispensaries from both regions were covered during this study. The scenarios and debriefing tool were piloted in three non-trial villages before data collection in June and July 2012.

**Table 1:** Mystery Client Scripted Scenarios and Debrief Checklists.

Scenario for Mystery Clients	Checklist for Mystery Client Debrief
**Information regarding STIs:** For a youth who is concerned about STIs after having had sex few days ago with a new partner. The youth did not use a condom during the sexual intercourse. His/her motivation to come to the health facility came from hearing from a friend that the partner has STIs. But the youth does not yet have any signs or symptoms. The youth is also worried that his/her parents would find out. The youth is sexually active and has had intercourse with more than one partner. S/he has very limited knowledge on the signs and symptoms of STIs. The only symptom the youth has heard of is genital itching.	*Empathetic welcome*?
	*Identification-name*, *age*, *address*, *marital status*?
	*Problem*: *signs and symptoms*?
	*Steps taken before going to the health clinic*?
	*When was the last sexual intercourse*?
	*Experience in condom use*?
	*Number of sexual partners*?
	*Any other health problem*?
	*How did the health provider receive you*?
	*Did you feel comfortable communicating with him/her*?
	*Did health provide give you advice on how to address your incident in future*?
	*Did health provider have an encouraging aura or was he/she negative*?
	*Did you feel ridiculed in any way by what the health provider told you*? *If yes*, *what did he/she say*?
	*Was there any negative comment directed to you or your appearance e*.*g*. *dressing*, *your age*, *your gender*? *If yes*, *which*?
	*Examination (request for examination*, *counselling on examination)*?
	*Counselling (share findings*, *reassurance*, *partner notification condom advice)*?
	*For females only (if asked about having children*, *use of any contraception*, *last menstrual period*, *about the history of lower abdominal pain during menses*?)
**Condom request:** The youth went to the health unit for condoms because s/he is sexually active. S/he has heard about STIs/HIV and that condoms may prevent them. The youth also heard that they are free at health units. However, the youth has heard many strange rumours about condoms including the notion that condoms contain HIV or that they have holes.	*Empathetic welcome*?
	*Identification*: *name*, *age*, *address*, *marital status*?
	*Have you ever used/seen a condom*?
	*Condom demonstrations*?
	*Condom provision*?
	*Correction of myths*?
	*Conclusion*?
	*Did you feel ridiculed in any way by what the health provider told you*? *If yes*, *what did he/she say*?
	*Was there any negative comment directed to you or your appearance e*.*g*. *dressing*, *your age*, *your gender*? *If yes*, *which*?
	*Did health provider have an encouraging aura or was he/she negative*?
	*Did you feel comfortable communicating with him/her*?
**General Family Planning:** 16 year-old school going youth visits the facility to obtain information on family planning services. The youth tells the provider that s/he has never had intercourse before. A schoolmate has been her/his girlfriend/ boyfriend for six months. Currently, the youth is feeling pressured by her/his girl/boyfriend to have sexual intercourse. The youth believes that if she/he does not accept s/he will leave her/him. S/he wants to avoid pregnancy to herself/his partner but knows very little about contraceptives. The youth has only heard of the pill and has many erroneous ideas about its use and possible side effects. The youth does not dare to talk with her/his parents about the problem, because they are very strict and would not appreciate this kind of relationship.	*Empathetic welcome*?
	*Identification*: *name*, *age*, *address*, *marital status*?
	*If asked for problem*?
	*If asked about last menstrual period*?
	*Recommendation from a service provider*?
	*Different contraceptives mentioned by the service provider*?
	*Detailed explanation concerning the different FP methods*?
	*Opportunity to choose the method preferred by the client*?
	*Explanation of the method chosen*?
	*Conclusion*?
	*Did you feel ridiculed in any way by what the health provider told you*? *If yes*, *what did he/she say*?
	*Was there any negative comment directed to you or your appearance e*.*g*. *dressing*, *your age*, *your gender*? *If yes*, *which*?
	*Did health provider have an encouraging aura or was he/she negative*?
	*Did you feel comfortable communicating with him/her*?

Mystery Clients (MCs) were blinded to the trial community status and in order to make sure that they were not identified by health workers they were dropped off one village away from the health facility to be visited. From this point, MCs walked, used bicycles or hired motorcycles to the facilities. As with previous MC evaluations [13] and to ensure accurate data capture, MCs hid digital recorders in their pockets. Immediately after their visits, debrief interviews were conducted and digitally recorded by trained research assistants using checklists. Male MCs were interviewed by a male researcher while female MCs were interviewed by a female researcher. All digitally recorded consultations between the patients and providers as well as debrief interviews with the MCs were verbatim-transcribed and translated. All the transcribed texts were read to identify the key emerging themes by the NIMR qualitative research team (ZS and SN). During the coding the team also explored predetermined themes, topics that were used to guide exit interviews with mystery clients themselves after completing the consultations. These themes and those that arose from the transcribed data guided the preparation of a preliminary report. The report was shared with an independent qualitative researcher (ER) who did not participate directly in the fieldwork. Alongside reading and commenting on the report ER selected a subset of transcripts (in total 36) by purposively sampling across both regions, intervention and comparison sites, types of ward and types of scenario to strengthen the credibility and confirmability of the findings. The team also discussed which quotes would most effectively illustrate the themes that had been identified and confirmed throughout the process of analysis.

### Ethical Considerations

*Int*HEC project was approved by the Liverpool School of Tropical Medicine Ethics Committee and by the Ministry of Health and Social Welfare through the Medical Research Coordination Committee. This study was included therefore under *Int*HEC project’s LSTM REC approval no. 09.75 and MRCC approval no. NIMR/HQ/R.8a/Vol.IX/1012. After the approval by the ministry, senior staff of *Int*HEC project met with regional and district authorities (in both Mwanza and Iringa) to request for assent to conduct the study in their region. Two months before the actual field work the officers in charge of health facilities were informed at a central meeting of the mystery client study, but the details of the dates and names of the specific health facilities were not provided to reduce potential bias from the Hawthorne effect (a phenomenon which refers to participants adapting their normal behaviour for reasons of social desirability). During the meeting with in-charges, *Int*HEC researchers orally presented information about the study, its objectives and procedures. Following clear understanding of the nature of the study, in-charges signed written consent forms stating their willingness for their respective facilities to participate in the research. This process was witnessed by respective District Medical Officers who attended the meetings. Thereafter, in-charges were requested to inform all health workers in their respective facilities about the study. No direct consent was obtained from the health workers as it was not possible to ascertain which health workers would attend young people during their visits to the facilities. However in-charges were asked to communicate with the research team if any of the health workers did not wish to participate and we did not receive any communication from in-charges on this matter. Confidentiality and anonymity of health workers were guaranteed by using codes in identifying health facilities during fieldwork and report writing.

Young people who eventually became our MCs provided written consent as follows: they were recruited in the capacity of research assistants and were provided with a copy of the study protocol that included an ethics statement, and signed a formal contract that included informed consent. On this basis, they received a standard field allowance for their training and during fieldwork. Parents were also informed of the terms and conditions that applied to their children’s participation in the study and were given an opportunity to ask questions about the study. Parents did not provide formal consent as the young people involved were all 18 years and above and able to provide their own consent. Although young people did not require parental consent, for cultural reasons we felt it was important to make parents aware of the study and the potential adverse outcomes to participation in case the MC identities were discovered; an event for which in this setting, the parents may be held accountable. For further protection, and once the field work started, MCs carried a signed letter of authorisation from their respective Regional Medical Officers in the event of their discovery by health workers.

## Results

Forty eight visits were made to 33 health facilities. No MCs were identified by any health service staff. Seven of the 48 digital recordings made during the consultations could not be deciphered and were excluded, giving a combined total of 89 transcripts from health worker consultations (41) and debrief interviews (48). Key themes emerged and are synthesised as follows:

### Inadequate or hostile reception

MCs reported a lack of clear signage or instructions in the majority of facilities visited (particularly dispensaries). In many cases, clients had to ask other patients where to access the services they required. One MC reported waiting three hours before being told on entry into the consultation room that she was in the wrong place. Youth specific waiting areas or signposting were entirely absent.

Health workers were often unavailable or facilities closed because of staff absences. Health workers often lacked motivation and professional demeanour. One nurse was found sleeping in the office and frequent lack of uniforms made other staff difficult for MCs to identify. Furthermore, receptionists were sometimes rude to young people:

“… *Where were you when I was calling your name*? *[…] Didn’t you hear your name*? *I was calling Lucia Marko*, *eeeh*? [pseudonym registered by the MC], *but you kept quiet*, *what does your behaviour mean*? *You make people sick and tired*, *are you the one who pays their salary*? *[…]*”

(Health worker, rural-intervention ward, Mwanza)

### Health worker attitudes to adolescent use of SRH services

In a number of cases across both regions, health workers displayed negative and paternalistic attitudes towards adolescent use of SRH services, including expressing the view that family planning should be reserved for adults, particularly married couples. This may have arisen from a common view that family planning services are largely meant for child spacing.

“…*When I started to explain my need of FP methods s/he asked me twice*, *Family planning*? *So*, *are you married*?

(Mystery Client, urban-intervention ward, Mwanza)

Knowledge and attitudes towards condom use among health workers were very variable. Whereas a number of health workers had positive attitudes to condom use, several expressed misgivings or openly discouraged young people from using them. For example:

“…*It is okay you can use condoms*, *but condoms do not protect someone from getting HIV infections because condom has got tiny invisible holes*. *Don’t say I have used condom*, *I will not get HIV*, *what we advise is that*, *it protects in a very small percentage*, *it protects pregnancy*, *sexual transmitted infections*, *but it doesn’t protect HIV […]*”

(Health worker, rural-control ward, Iringa)

Conversely, health workers also spoke out against this kind of attitude:

“…*Those are lies*, *people speak lies*, *had it been true*, *the government would have noted it because all the items imported are approved before being supplied*, *they don’t supply without approving them*, *people fight against AIDS […]*”

(Health worker, special risk-control ward, Iringa

In addition there were unfortunately several examples of unprofessional behaviour by health workers. One laughed at a young person when he admitted to a lack of knowledge about condoms, and MCs at times complained of hostile and judgmental health workers who made them uncomfortable. MCs also expressed discomfort at being asked questions about themselves and their complaints in the presence of strangers in waiting rooms or open offices.

Even when attitudes to youth access were positive, health workers sometimes demonstrated discriminatory gender stereotypes:

*“....as for a woman*, *you shouldn’t have multiple partners*. *As for men*, *it’s not easy to abstain*, *for example; he can’t stay in Dar without having another partner*… “

(Health worker, rural-intervention ward, Iringa)

*“If you let her use pills she will be engaging with sexual affairs with other guys*. *For someone who is not your wife do not take pills as she will use them and sleep with other people [men]*. *The time she sleeps with other people*, *you will not realize*. *There are STIs and HIV*, *do not use that one [Pills]*!! *Now*, *in protecting yourself [form STIs]*, *just take condoms*, *understand […]*?”

(Health worker, special risk-control ward, Iringa)

### Lack of privacy and confidentiality

In a significant proportion of cases (19 out of 48), mystery clients complained of inadequate infrastructure, or efforts made, to protect their privacy. In some health facilities, consultation room windows were reported to be open and facing the waiting rooms. In addition, MCs were attended to while other patients were in the room. There were a number of examples of this lack of privacy, partly due to the infrastructural challenges and partly due to health worker breaches of confidentiality. The following mini-scenarios illustrate some of the key issues encountered by young people:

- Two clients regardless of their different age and or gender attended concurrently by two health workers in the same room;
- Consultation with adolescent clients took place in a corridors or waiting lounge in presence of other people;
- Adult clients were sometimes allowed to enter the consultation rooms before the health providers had finished with the preceded patient;
- In two health facilities, service providers invited their colleagues to enter the consultation room to listen the conversation between the provider and the clients and/or staff members entered the consultation room without notice;
- In another health facility service providers were heard discussing about the health complains of their previous clients, immediately after their clients had left the room.

In most cases and particularly at dispensary level, consultation rooms were located close to waiting rooms. Moreover the windows and/or doors of these facilities were kept open and therefore, where two MC clients were sent to one facility (to perform different scenarios), the succeeding mystery client could easily hear the conversation between her/his colleague and the service provider. The following quote is illustrative of the combined challenges of the physical constraints to privacy, exacerbated by the service provider decisions that further reduced privacy and how this impacted on MCs sense of the confidentiality of their consultations:

*“…That room [consultation room] lacked privacy*. *Because on the chairs*, *when I got in*, *other women came*, *about four of them*, *they were standing at the door waiting to see the same doctor*, *and then another doctor came and got in to that room*, *I think two doctors use to stay in that same room while attending clients…*”

(Mystery Client, special risk-control ward, Mwanza)

### Logistical challenges

Several facilities, especially the dispensaries had no STI treatment services. Furthermore, where STI treatment was available, young people were usually charged for their use. In Tanzania, although services for STIs are supposed to be given for free (under the community health insurance scheme) there are often minimal charges that people have to pay when they visit facilities. Thus, young people ended up having to pay fees for services that should have been free, for example for a registration card or a consultation fee. In addition, due to drug stock-outs, patients are often advised to purchase medicines for drug shops to treat STIs, mostly antibiotics in capsule form which in Kiswahili are nicknamed *rangi mbili* (meaning two-coloured medicine).

Lack of an appropriate working schedule in provision of family planning services was also an important barrier toward access of services to adolescents. Although government facilities are required to operate from 7:30 am to 3:30pm, Monday to Friday, facilities often closed early. Additionally, in most health facilities visited, family planning was only available in the morning, making access for those attending schools particularly difficult.

### Good practice

Across Mwanza and Iringa regions, we found that that the strongest themes emerging from the data illustrated the challenges explored above. However in a smaller number of cases, across both regions the study also revealed some very good practices including support for condom use, sympathy at expressed symptoms of STI and encouragement to bring other young people to the service, as in the following examples:

“… *We are always singing [insisting] that you use condoms to protect yourself*. *Now everyone should do it [use condoms]*. *This is because you cannot test someone by merely looking at him*, *she might be looking smart but only to find that she is already infected [with HIV]*, *guys use condoms*, *stop being careless*, *how long is it since you had sex with her*…”

(Health worker, rural-control ward, Mwanza)

*“Would you please bring many others who are in need of advice so that they get counselling services hence protect themselves from STIs*, *HIV/AIDS as well as early and unplanned pregnancies […]*”

(Health worker, rural-intervention ward, Mwanza)

Finally, while we did not undertake a rigorous evaluation of the MC experiences of carrying out the research we asked them to reflect on the research process and explored whether they would be willing to participate in future studies. Our young, trained mystery clients reflected positively on their experiences of being involved in the study and expressed willingness and enthusiasm for getting involved in future research.

## Discussion

We conducted a large scale mystery client evaluation of the youth friendliness of government health facilities in two districts in Mwanza and Iringa Regions in Tanzania. It was clear from our findings that negative health worker attitudes, coupled with infrastructure constraints and weaknesses, remain significant barriers to young people’s access of sexual and reproductive health services across Mwanza and Iringa.

Adolescents in rural and urban low resource settings are among the most vulnerable in society, often facing restricted educational and employment opportunities [15]. Additionally, in societies where greater age conveys power, young people experience constrained agency, especially in relation to sexual and reproductive health [2, 4]. Our study confirms the existence of age and agency related constraints in young peoples’ negotiations with health workers around sexual and reproductive health issues in the context of two regions in Tanzania. Furthermore the findings reveal the existence of gendered power relations, illustrated by a small yet significant number of cases that impact negatively on young women’s ability to negotiate sexual and reproductive health questions with older male and female health staff [4].

We found that dispensary and health facility infrastructure present varying levels of barriers, from those which could be fairly easily addressed (e.g. signage and ensuring staff use uniforms or other identifiers such as badges or labels to indicate their role) to those infrastructural issues which have long been recognised as challenges for the Tanzanian health system (e.g. fit-for-purpose buildings with appropriate receptions that are attached to waiting rooms, with sufficient numbers of separate and ‘closed door’ consultation rooms) [16].

In particular, our findings highlighted that lack of privacy and confidentiality impacted significantly on our mystery clients’ views on the appropriateness and acceptability of the services they encountered. Evidence from other baseline studies conducted during the IntHEC programme suggests that adolescents may be more likely to seek services from close-to-community providers such as local ‘drug shops’ and, in some cases, traditional healers [17]. Similar findings from other settings suggest that the use of traditional healers may be linked to perceptions that these services provide greater privacy and confidentiality for users [18]. Services for young people need to be equitable, accessible, acceptable, appropriate and effective [1]. The quality of these services relies on each of these components being fulfilled [19].

The study we undertook provided important illustrative evidence that serious challenges remain despite long-term investments of the Tanzanian Ministry of Health in adolescent-friendly services. These challenges not only require continued long-term financial resources, but also sustained advocacy to address persistent social and cultural norms underpinning discriminatory attitudes towards young people and adolescents seeking sexual and reproductive health services. A recent estimate suggests that relatively modest increases in investment (less than 1% of overall health expenditure) to improve quality health service delivery to adolescents would lead to exponential gains for adolescent health in Africa [19]. However, data are lacking on the approaches and resources required to transform socio-cultural norms influencing the attitudes of health care workers and other significant gatekeepers of adolescent health [17].

Overall our findings suggest that serious challenges remain for improving Tanzania’s adolescent sexual and reproductive health services. In terms of infrastructure, action is needed to increase visibility of youth friendly services and to increase privacy for all clients of health services, not just young people. Since some health workers were found to display positive and supportive attitudes towards young people it could be appropriate to investigate these cases of ‘positive deviance’ to understand more about what contextual and individual factors promote these kinds of attitudes among health workers. This could help to develop existing education and training curricula on youth friendly services for health workers.

### Methodological constraints and considerations

An important limitation of our work is that for cultural reasons the MCs could not ask the health workers their names as this would have been unusual in the local setting and would have raised suspicion as to their identity and would have jeopardised the research. We were therefore unable to assess the proportion of health workers involved in this study who had actually received youth friendly services training.

The tone and language used in an interaction between health worker and client is critical to the quality of the interaction. This is particularly so in the case of adolescents. Yet capturing this is extremely difficult. Our ability to review verbatim transcripts of both the consultations and the MCs’ immediate reactions improved the rigour and depth of our evaluation. The size of the study and inclusion of a range of communities further improves the credibility of the findings.

The ethical challenges of MCs adopting different persona to approach health workers, were largely addressed by the briefing of staff well in advance of the study. For their part, the MCs were exposed to negative attitudes which could have led to anxieties at the time, however it was clear from debriefs that their thorough training coupled with the research team’s support of MCs during fieldwork allowed young people to appropriately deal with their experiences. In fact, they expressed considerable satisfaction in relation to their role in the study. This supports findings from other studies that ‘mystery client’ or ‘simulated patient’ methods offer feasible and acceptable opportunities to explore health worker-client interactions in this context [10, 13].

Finally it is important to note that whilst we have disseminated the findings of this study at national conferences attended by policy makers and programs officers from the regions and Ministry of Health and Social welfare, the names of the participating facilities and their respective staff were strictly anonymized. Shortly before carrying out a follow-up MC study (in early 2014) we gave very preliminary feedback to the District Medical Officers, but we also strictly anonymised the names of the facilities and health workers who participated in the baseline (MC) study to ensure that the DMOs could not identify the individuals or facilities, to avoid any repercussions for them. The dissemination to the health workers from the intervention and control arms is scheduled to take place once the analysis of the final MC study is completed (the IntHEC study ended at the end of October 2014 and the final stage of data analysis and write-up is on-going). We will continue to anonymize the names health workers who participated in the study and will not link facilities to any specific finding at future dissemination events.

## Conclusion

The need to develop youth friendly services, especially in the area of sexual and reproductive health, has been recognised and promoted as a priority both globally and in the specific context of Tanzania. Our investigation of these services through the use of mystery clients found that challenges and barriers remain due to unhelpful and judgemental health worker attitudes towards young people and due to infrastructure and logistical constraints. While it is clear that there is still a long way to go in Tanzania’s development and implementation of youth friendly sexual and reproductive health services, there are opportunities to learn from cases of good practice among health workers to identify the contextual or individual reasons for their positive attitudes.
